# Diversity awareness, diversity competency and access to healthcare for minority groups: perspectives of healthcare professionals in Croatia, Germany, Poland, and Slovenia

**DOI:** 10.3389/fpubh.2023.1204854

**Published:** 2023-07-21

**Authors:** Mojca Ramšak, Marcin Orzechowski, Katarzyna Bielińska, Anna Chowaniec, Robert Doričić, Marianne Nowak, Tobias Skuban-Eiseler, Ivana Tutić Grokša, Paweł Łuków, Amir Muzur, Zvonka Zupanič-Slavec, Florian Steger

**Affiliations:** ^1^Faculty of Medicine, Institute for History of Medicine, University of Ljubljana, Ljubljana, Slovenia; ^2^Institute of the History, Philosophy and Ethics of Medicine, Ulm University, Ulm, Germany; ^3^Faculty of Philosophy, Center for Bioethics and Biolaw, University of Warsaw, Warszawa, Poland; ^4^Department of Social Sciences and Medical Humanities, Faculty of Medicine, University of Rijeka, Rijeka, Croatia; ^5^Department of Public Health, Faculty of Health Studies, University of Rijeka, Rijeka, Croatia

**Keywords:** access to healthcare, healthcare inequality, health services, cultural competency, public hospitals, ethics, diversity, disadvantaged population

## Abstract

**Introduction:**

Due to cultural, language, or legal barriers, members of social minority groups face challenges in access to healthcare. Equality of healthcare provision can be achieved through raised diversity awareness and diversity competency of healthcare professionals. The aim of this research was to explore the experiences and attitudes of healthcare professionals toward the issue of social diversity and equal access to healthcare in Croatia, Germany, Poland, and Slovenia.

**Methods:**

The data reported come from semi-structured interviews with *n* = 39 healthcare professionals. The interviews were analyzed using the methods of content analysis and thematic analysis.

**Results:**

Respondents in all four countries acknowledged that socioeconomic factors and membership in a minority group have an impact on access to healthcare services, but its scope varies depending on the country. Underfunding of healthcare, language barriers, inadequate cultural training or lack of interpersonal competencies, and lack of institutional support were presented as major challenges in the provision of diversity-responsive healthcare. The majority of interviewees did not perceive direct systemic exclusion of minority groups; however, they reported cases of individual discrimination through the presence of homophobia or racism.

**Discussion:**

To improve the situation, systemic interventions are needed that encompass all levels of healthcare systems – from policies to addressing existing challenges at the healthcare facility level to improving the attitudes and skills of individual healthcare providers.

## 1. Introduction

Addressing the healthcare needs of minority groups becomes one of the major challenges of modern healthcare systems in Europe. Research on the topic indicates that several minority groups are at risk of being underserved or receiving suboptimal services (1). Belonging to a social minority can be an obstacle to receiving timely and appropriate healthcare (2). For example, migrants and refugees experience disparities in accessing healthcare due to legal, language, or administrative barriers (3). Deprived, in many cases, of adequate healthcare in countries of their origin, susceptible to illness and physical as well as psychological abuse or trauma *en route*, migrants often belong to vulnerable groups and require special attention with respect to the provision of healthcare. Another disadvantaged group is constituted by non-heteronormative persons, such as gay, lesbian, and bisexual persons as well as transgender and non-binary gender individuals, who report substantial discrimination, stigmatization, and disparities regarding their access to healthcare (4, 5). These stem from institutional and/or internalized forms of homophobia, such as healthcare providers’ moral convictions and attitudes (6, 7). Inequalities also affect other minority groups, e.g., religious groups (8), individuals with disabilities (9), or older people (10).

The issue of equal access to healthcare is central from the point of view of both public health ethics and medical ethics. Moral legitimacy of equal access to healthcare stems from the essential role that health plays in human flourishing through maximizing the health of the population or individuals and the promotion of equal access to healthcare as a part of a broad conception of social justice (11). Equality of access to healthcare involves the medico-ethical responsibility to minimize differences in providing the best healthcare for individual patients, regardless of the social minority groups to which they belong. This should occur in congruence with the medico-ethical principles of justice, autonomy, beneficence, and non-maleficence (12).

Cultural awareness and cultural competency play an important role in access to healthcare. “Cultural awareness is an understanding and knowledge of a relevant cultural issue that is not always accompanied by a common or acceptable practice or action” (13, p.135); whereas cultural competence is: “A set of consistent behaviors, attitudes, and policies that come together in a system, agency, or among professionals to allow that system, agency, or profession to work effectively in cross-cultural situations” (13, p. 135). Thus, cultural competence encompasses a commitment to appropriate policies and practices to improve the capacity for the provision of healthcare for diverse populations (14). “Cultural competence is much more than awareness of cultural differences, as it focuses on the capacity of the health system to improve health and wellbeing by integrating culture into the delivery of health services. It involves understanding and integrating differences and incorporating them into daily care and working effectively in cross-cultural situations” (13, p. 135). This approach includes various interventions that among others could include a diverse composition of healthcare staff, provision of cultural training, and better access to interpreter services as well as the improved stance of healthcare professionals towards care for diverse groups of patients (15). A key role in the provision of diversity-appropriate care is played by healthcare professionals as they serve as the main point of contact in medical encounters. Inadequate diversity competence can lead to misunderstanding, patient mistrust, and lower satisfaction with healthcare on the part of patients (16, 17). Although cultural awareness and cultural competence are terms used in up-to-date literature on the topic, they narrow the understanding of the personal or institutional capacity to provide equal healthcare to racial, ethnic, or religious minority groups. Therefore, in our research, we use the term ‘diversity competence’ (18) to include also other minority characteristics such as a non-heteronormative sexual orientation or gender identity.

Addressing the issue of inequality in access to healthcare requires not only an understanding of the patient’s perspectives but also of the perspectives of healthcare professionals. Therefore, the aim of this research was to examine the current situation concerning access to healthcare from the point of view of actors in the healthcare space regarding the issue of social diversity and equal access to healthcare in four European countries: Croatia, Germany, Poland, and Slovenia. These countries were selected for several reasons. First, although all four countries are Member States of the European Union and through this subject to European regulations concerning equality in access to healthcare, their national policies vary in regard to protection from discrimination in healthcare (19, 20), which has direct consequences for diversity competency of healthcare organizations in these countries (21). Moreover, they vary under socio-cultural aspects. Germany has been a migration destination country for several decades now, whereas Croatia, Poland, and Slovenia only recently became migration transition or destination countries. Therefore, religious or cultural differences in these three countries are not as prominent as in Germany. Third, they differ with regard to their levels of economic development, which has direct consequences for allocation of resources in healthcare and introduction of specialized programs towards diversity competency. In addition, insufficient number of healthcare professionals in Germany in recent years led to increased number of foreign workers in the healthcare sector in this country and, consequently, changes in socio-cultural structure of healthcare workforce.

These countries can also be compared with regard to organization of their health insurance systems. In Croatia, Poland, and Slovenia, health insurance is centralized, while Germany has a more decentralized and complex system with a free choice of providers. All countries have an obligatory social health insurance system (SHI) that results in nearly universal coverage for residents. Supplementary voluntary health insurance (VHI) can be purchased to cover co-payments in Croatia, Germany, and Slovenia. VHI in Poland can cover healthcare services provided outside the public system by private companies, whereas medicines and medical goods are co-paid (20). The core values of all systems are universality, solidarity, equality, equity in funding, accessibility, quality, and safety (22–25).

Moreover, there is a difference between these countries regarding legal protection against discrimination in healthcare, which has been discussed in detail previously (20). In all four countries, constitutional-level legislation guarantees the right to healthcare and protects against discrimination. However, national laws and regulations prohibiting discrimination in healthcare based on factors other than race and ethnicity vary widely. There are differences between Croatia, Germany, Poland, and Slovenia with respect to characteristics such as sexual orientation, gender identity, religion, and belief. Poland lags behind the other three countries in terms of the completeness of relevant legislation, which is most prevalent in Slovenia and Croatia, and then in Germany. Despite the fact that equality principles are explicitly enshrined in European Union treaties and national constitutions, members of minorities may face barriers to accessing healthcare services (20). Therefore, the issue of equal access to healthcare for various minority groups can be identified and recognized differently in each of these countries. In our study, we focused mainly on three dimensions of diversity: (i) race and ethnicity; (ii) religion and belief; and (iii) gender identity and sexual orientation.

## 2. Materials and methods

### 2.1. General

For the purpose of this research healthcare professionals and representatives of hospital staff from Croatia, Germany, Poland, and Slovenia were interviewed. For this research, a qualitative research design in the form of explorative narrative interviews was used. The purpose of the interviews was to gain insight into the subjective perspectives of the interviewees. The interviews were semi-structured. The formulation of the interview questions and the setting of the focal points serve to provide statements within the context of the interview topic and the backgrounds of the interviewees. Exploratory interviews give the flexibility to ask *ad hoc* questions to clarify statements or focus on specific issues. This type of interview includes expert questions, narrative segments, and focus segments with narrative characters (26). The interview questions were prepared on the basis of a literature review and results of the previous research conducted by the team of authors (19–21). Moreover, questions of validity and possible bias in the results were extensively discussed by the team of authors.

### 2.2. Design of the empirical investigation

A multi-professional team of authors from all four countries under investigation, with backgrounds in medicine, public health, ethics, history of medicine, philosophy, ethnology, anthropology, political science, and social work, designed the investigation. The interviews were conducted by eight authors (two from Croatia and two from Poland, three from Germany, and one from Slovenia), seven of whom have PhDs. One researcher is a Ph.D. student. All interviewers had knowledge of the research topic, qualitative research methods, and formal qualifications or experience required for interview organization and conduct. The interviewers were fluent in the language used for the interviews.

Purposive sampling was used in the research design to select participants who could provide answers pertinent to the research topic (27). First, selected healthcare institutions that acted as gatekeepers or potential participants with whom personal contacts already existed were contacted directly. Healthcare institutions were contacted via letters or emails. Representatives of these institutions forwarded information about the research to the employees of the institutions/potential participants. The first contact with the potential participants was made via post or email or telephone and included information about the research’s purpose, method, and the institution conducting the research, as well as the ethical aspects of research (voluntariness of participation, possibility of withdrawal, and confidentiality). Participants who expressed their willingness to participate in the research were scheduled for an interview, either face-to-face or remotely.

For the interviews, we created a set of questions in English that were the same for all four countries and were tailored to the cultural and systemic characteristics of health systems in each country. The questions focused on ethical issues that resulted from the challenge of diversity in hospital healthcare practice, particularly: (a) the discrimination awareness and discrimination experience in the access to healthcare, (b) systemic and cultural barriers to accessing healthcare, (c) knowledge of legal and institutional norms regarding equal access to healthcare, and (d) suggestions for improvement of the situation. The interview questions were then translated into each country’s official language, i.e., Croatian, German, Polish, and Slovenian. Ethical approval of the research was provided through institutional research ethics committees at each institution participating in the research.

### 2.3. Conduct of interviews

We conducted 39 interviews with healthcare professionals in Croatia (*n* = 10), Germany (*n* = 10), Poland (*n* = 7), and Slovenia (*n* = 12). The representatives of the following professions were interviewed: medical doctor, nurse, hospital manager, lawyer, economist, social worker, midwife, psychologist, medical interpreter, and paramedic. The interviews were conducted from July 2021 to March 2022. In Poland, all the interviews were conducted before the February 24, 2022, that is before the aggression of Russia against Ukraine, which was followed by enormous changes in healthcare, including legal changes. Due to the Covid-19 pandemic control measures at the time of the interviews, remote interviews were considered necessary in some cases.

The interviews were carried out face-to-face or by using a secure communication platform. The online interviewing method allowed participants to communicate without being at risk of contracting Covid-19 (28). Interviews were conducted in Croatian, German, Polish, Slovenian, or English. Individual interviews lasted between 40 and 180 min. Interview transcripts were not returned to participants for comment or correction. Repeated interviews were not conducted.

Prior to the recruitment process and at the start of the interviews, the interviewees were given information about the project’s goals. Respondents were informed in writing about the purpose of the research, the voluntary nature of their participation, the anonymization of their data, the protection and archiving of the data obtained, that the interview would be recorded, and that the research consortium intended to publish the results of the interviews with anonymized responses. Interviews were conducted with the individuals who gave their consent to it. The interviewees took part in the research on a completely voluntary basis and were not compensated in any way. The interviewees had the option of refusing to answer specific questions or terminating their participation at any time without negative consequences, and they could request that the personal data collected to destroyed. However, this option was not used by any of the participants. Because the research questions do not specifically study the influence of individual characteristics of interviewees, i.e., age, gender, career path or professional background, on the research topic, no demographic data have been collected during the interviews.

### 2.4. Data analysis

Interviews were digitally recorded and transcribed by the researchers conducting the interviews or by external transcription service; after this stage, the data of the participants were anonymized. Each national research group in the consortium analyzed the data from the transcribed interviews conducted in their country, in the language in which the interview was recorded. Data analysis was conducted using the methods of content analysis (29) and thematic analysis (30, 31). First, the responses of the interviewees were reduced to core elements and statements. These elements were manually coded, extracted, and systemized through clustering into main topics and subtopics. These topics were inductively formulated based on the content of the interviews, in order to identify important and recurring themes and differences between responses. After that, the researchers translated all of the codes into English. Representative quotes were translated into English.

## 3. Results

To understand the importance of diversity awareness and diversity competence in relation to persons who experience difficulties in access to healthcare in public healthcare facilities from the perspective of healthcare professionals, we summarized their interpretations and understanding of the situation in several key thematic areas.

### 3.1. Persons who experience difficulties in access to healthcare

With regard to persons who experience difficulties in access to healthcare, respondents acknowledged that socioeconomic factors and membership in a minority group has an impact on access to health services. Common groups that were identified in all four countries were race and ethnic, national, cultural, and religious minority groups, migrants and foreigners. They can experience barriers in access to healthcare due to administrative difficulties, limitations in communication with healthcare professionals, various cultural perceptions of illness and health, or lack of knowledge of healthcare systems. Moreover, as disadvantaged in healthcare system also representatives of marginalized social groups, such as people in homelessness crisis, unemployed persons, persons with alcohol dependence or substance abuse were identified. It was mentioned that these persons often lack the assistance of family or friends, have reduced social contacts and thus have difficulties in entering and navigating the healthcare system.

*So, vulnerable groups in society and in the health system are generally groups that are also vulnerable in other categories. These are older people. These are people, who are powerless due to illness or some kind of disability* (Croatia).

**Figure 1:**
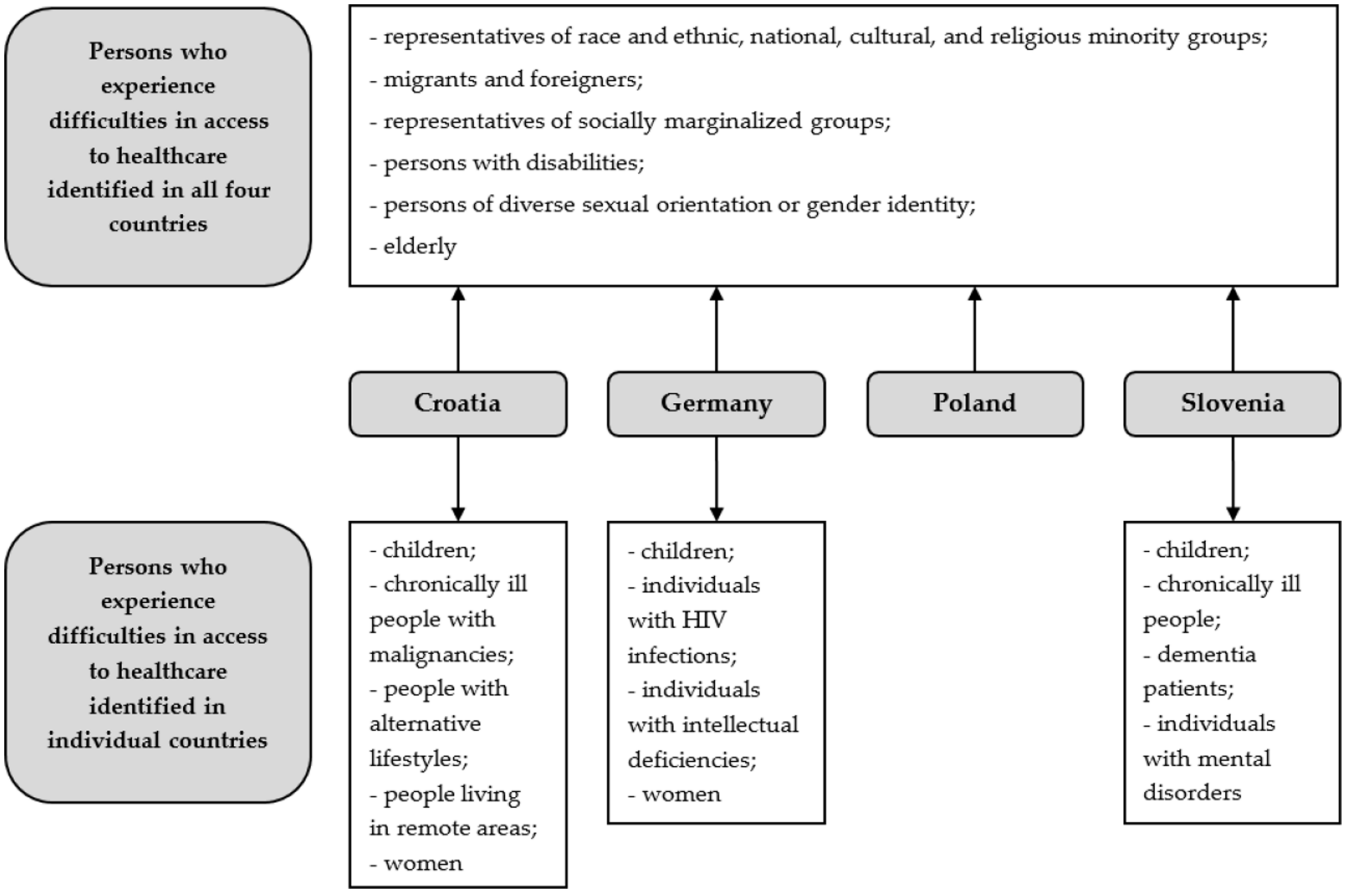
Persons who experience difficulties in access to healthcare identified by interviewees in Croatia, Germany, Poland, and Slovenia.

Furthermore, in all four countries we identified people with disabilities and older adults who experience difficulties in access to healthcare, mostly due to their dependence on other persons (Figure 1).

Among the groups that were additionally identified by the respondents in individual countries, the interlocutors in Croatia reported persons with alternative life-styles, people living in rural, geographically isolated areas, or chronically ill people with malignancies, especially during the Covid-19 pandemic.

*In our case, it turned out that vulnerable people are those who are geographically isolated, that is, who live in rural areas, because the service is quite inaccessible to them* (Croatia).

Interviewees in both Croatia and Germany pointed to women as disadvantaged group. Gender factor can in some situations lead to double marginalization in the healthcare system; first, because women are vulnerable to marginalization in general, but especially when it is associated with other types of marginalization, such as belonging to an ethnic or religious minority.

Polish professionals pointed out that the main challenge of healthcare in Poland is underfinancing, which leads to long waiting lists, and shortages of resources. This is the main barrier to access, which affects the general population. However, they observed that some groups experience specific difficulties. Identified were medical staff’s discriminatory attitudes, stereotypes, and prejudices towards patients of diverse sexual orientation or gender identity, although some progress in recent years has been observed in this respect. Transgender persons’ treatment is still a challenge, also on the institutional level, due to the lack of guidelines and financial and infrastructural conditions of hospitals.

Similarly, in Slovenia, individuals of non-heteronormative gender identity or sexual orientation were identified as disadvantaged. Moreover, named were chronically ill, regardless of age, with incomes that do not allow for adequate coverage of basic living needs, who rely on the help of relatives and cannot afford to pay for adequate care at home or in nursing homes. Several other groups have also been mentioned, such as dementia patients, children from socially disadvantaged families, who are often not identified because they do not disclose their poverty for fear of stigmatization, persons who have experienced domestic violence, or women who have a special status within their home in terms of being dependent on their male relatives, such as some Roma and some Muslim women. Women from Kosovo, who usually are absent from the labor market and do not speak Slovenian, were perceived as being particularly at risk of difficulties related to access to healthcare. Interviewees in Slovenia did not consider Roma people as particularly disadvantaged in general, mostly due to the fact that they receive good healthcare, and healthcare professionals are aware of their special cultural needs.

### 3.2. Presence of discrimination

In general, respondents in Croatia, Germany, and Slovenia, believed that there was no systemic discrimination in access to health care from a normative perspective; evidence that Polish respondents shared this view has not been obtained. The majority of interviewees in Croatia pointed out that, principally, there is no discriminatory behavior towards minorities in healthcare institutions. The participants from Croatia did not rule out the possibility of misunderstanding the concept of diversity among some of health workers. But, they also point out that hospital regulations generally prohibited discrimination in relation to all personal circumstances.

*And now, as far as minorities are concerned, I really did not notice that in the hospital we somehow also have minorities, but it does not come to the fore as much because that part always falls into the background. That priority is always illness* (Croatia).

Similarly, in Germany, respondents acknowledged that there was no structural discrimination in access to healthcare for minorities. However, they also mentioned that inadequate healthcare structures affect the care of minorities, especially migrants or persons of diverse sexual orientation or gender identity.

Discrimination occurs on individual level due to language or cultural barriers, personal attitudes time pressure, or lack of specific training.

*For me, vulnerable groups in healthcare are these, which have difficult access because they experience discrimination. It is so in case of transsexual persons or persons with HIV. Up to this day there is a discriminating behavior towards them in healthcare. LGBTQI persons in every form and extent* (Germany).

Polish healthcare workers pointed out that a possible systemic barrier to access to healthcare services was due to a lack of relevant regulation and legislation, both at the hospital level and the healthcare system in general. Here, they pointed primarily to issues related to the lack of insurance and the underfunding of healthcare, which affect various aspects of hospital work. This resulted in the inability to provide adequate care for patients from minority groups, such as the most frequently indicated problem with the lack of interpreters.

Respondents in Slovenia noted that discrimination in healthcare did not exist in principle and is prohibited in legal and hospital documents. In practice, however, there are both systemic and personal deviations from this principle. The general opinion is that access to public healthcare is good, except for the queues that cause patients to wait unreasonably long for some specialist examinations at the secondary level, and there are also long queues at the primary level, for example in dentistry or gynecology, except in urgent, life-threatening cases. Therefore, if a member of a disadvantaged group is waiting in line, it is not because of his or her personal circumstances, but because the healthcare system does not function optimally in this regard.

### 3.3. Presence of discriminatory behaviors

Interviewees in all four countries admitted that instances of discriminating behavior towards patients existed in individual cases (Table 1).

**Table 1:** Identified examples of discriminatory behavior towards particular minority groups.

	Target groups	Examples of individual discriminatory behavior
Croatia	Persons of diverse sexual orientation or gender identity	Gossip Inappropriate communication Stereotyping Stigmatization
	National, ethnic, and religious minorities	
Germany	Persons of diverse sexual orientation or gender identity	Gossip Stereotyping Stigmatization Less friendly behavior Inappropriate communication
	National and ethnic minorities	
	Migrants and asylum seekers	
Poland	Persons of diverse sexual orientation or gender identity	Stereotyping Gossip Stigmatization Inappropriate communication Discriminatory attitudes among other patients
	National, ethnic and religious minorities	
Slovenia	Persons of diverse sexual orientation or gender identity	Stigmatization Inappropriate communication
	National and ethnic minorities	
	Migrants and asylum seekers	

Examples of such behavior include gossip about same-sex partners during hospital visits or jokes about a patient’s health condition that is perceived as a result of their sexual orientation. Homophobia by patients toward persons of diverse sexual orientation or gender identity is also frequently noted, as well as general lack of knowledge on specific cultural needs.

*Of course, there were employees who had no tact and who were perhaps more aggressive toward someone, less tolerant because someone is gay and he does not like gay people. That exists, but I have not experienced it. I heard those stories from conversations* (Croatia).

In Germany, according to our interlocutors, stereotypes about minorities and their susceptibility to certain diseases, e.g., homosexuals and HIV infections, do occur. The issue of discrimination was also mentioned in the case of persons of diverse sexual orientation or gender identity in the context of various treatments, such as at the dentist or general practitioner, even if professional ethics prohibit discriminatory behavior. Interviewees mentioned, that when such behavior is present, it consequently affects how individual patients are treated, even though it is not expressed directly to patients. Concerning persons of diverse sexual orientation or gender identity, interviewees from Poland recalled some discriminatory behaviors. The interviewees themselves declared that sexual orientation in principle was an issue out of the scope of their medical interests. However, they perceive the treatment of transgender persons as a practical challenge in Polish hospitals. The concern of potential discriminatory attitudes among other patients was also expressed:

*It was a big problem, which we also had, what room she should be admitted to. In fact, it was quite a big problem. In our case, it solved itself in a very simple manner, because there were so many patients, that she was in the corridor. As many persons sometimes have to be (there). It should not be like that, it is scandalous that it is like that, but what room she would be admitted to (if there were any available beds in rooms)? It is a difficult question because a couple of times I heard such a story, similar stories. The problem is that it first depends on what the patient has written on the ID (…). How big are the rooms? If I had a choice and an opportunity to admit the patient for example to a single room, I’d try to do it, because it is also about other patients (…) who could have some problems (with it), I do not know, comments and simply to wrangle (…)* (Poland).

In several interviews, respondents from Croatia, Germany, and Poland mentioned specific examples of less friendly or even discriminatory behavior towards national and ethnic minorities, migrants, and asylum seekers. Polish professionals declared that they tried to treat all patients equally in their practice. They also said that unequal treatment could result from an employee’s personal biases against people with a different worldview from their own, patients of minority characteristics to whom they have a negative attitude, or people who do not fit the social norms. Polish healthcare workers admitted that some of their co-workers sometimes exhibited negative behaviors based on stereotypes. Negative attitude to religiously motivated decisions, which are sub-optimal for health, was also mentioned and one interviewee concerned it discrimination. Negative stereotypes toward people from minorities mostly translated into hurtful language, mostly used not in conversations with the patient but behind their backs, in conversations with other members of staff.

*We do not do any harm to him, God forbid, but we are chattering on him in the duty room* (Poland).

*No, rather, such a patient was also supplied in the emergency room, I think in a proper manner, for various reasons proper, but proper, right? Another thing was that when the door closed behind him, curses flew very often* (Poland).

The interviewees from Poland declared that negative stereotypes were a personal problem for individual employees but it did not translate into inferior treatment in practice. They also observed progress in attitudes to diverse patients in recent years. They pointed to possible differences between hospitals in large cities, where contact with people from minority groups is more frequent and thus employees are better prepared to care for them, as opposed to hospitals in smaller towns.

In Slovenia, sexual minorities face some discrimination due to a lack of medical specialists, for example, there is only one interdisciplinary Council for Gender Identity Confirmation, in the entire country, which functions as a gatekeeper, and some gender reassignment surgeries are not available in the public system because of lack of specialists. As a result, many transgender individuals choose to use self-paying surgical and psychiatric services abroad or in private clinics in Slovenia when they are under stress, buy hormones on their own on the Internet, and are generally dissatisfied with the legal and medical aspects of the transition process, finding it too lengthy and humiliating. For example, the guidelines for procedures for medical confirmation of gender identity are not publicly accessible, and only as of October 2022, transgender individuals in Slovenia can apply to any administrative units to change the gender information on their identification cards, reducing the possibility of involuntary disclosure of their transgender status, which could subsequently lead to stigma and discrimination. Before, only certain administrative units took measures to ensure the confidentiality of gender change procedures. In addition, the staff who treat transgender persons do not see their changed gender status on health cards, which leads to uncomfortable and humiliating situations and sometimes inappropriate communication, which is why transgender people have to reveal themselves, even when they do not want to. Some doctors also treat them as curiosities, sometimes not knowing exactly what to do with them in terms of communication, or, in the worst cases not wanting to treat them at all, informally referring to conscientious objection. Medical personnel is generally not adequately trained in communicating with sexual minorities, and unless treatment involves reassignment procedures, they act on their experiences and expect patients themselves to tell them how to address them or what form of communication is acceptable to them.

*Members of non-normative gender identities and sexual orientations, in my opinion, make very little use of health services, especially mental health services. This is probably because they fear that their behavior will be pathologized, while at the same time, they fear that medical experts will determine that they may have mental health problems. Medical experts treat men and women very differently. This is reflected, for example, in stereotypes into which they insert their own unmet needs; in the case of homosexuals, they share that their experiences are extremely foreign to them, that they find them unusual, and that they wonder about their “specific life choices.” The professional director of our hospital, during medical reports, went on whole tirades about how the parents of homosexual children should behave and how tragic it is not to be able to treat these tragic conditions* (Slovenia).

*A man who was in transition came to me in the course of hospital treatment and expressed the distress he felt because of the doctor’s treatment. During the conversation with the doctor, he reportedly felt that the doctor did not like him, he doubted that he was being treated properly, and he emphasized the shame he experienced during the visit along with his fellow patients. He did not want the matter to be brought to the doctor* (Slovenia).

### 3.4. Language barriers

In all four countries, language barriers were primarily mentioned as a major systemic shortcoming. The interviewees pointed to several similar shortcomings, such as the use of *ad hoc* solutions, lack of trained medical interpreters, and lack of money to hire them, which can lead to suboptimal quality healthcare.

*The problem is mostly language communication (…). Our employees mostly speak English, and there is always someone on shift who speaks English well, but a little less in the medical sphere, and if some other languages are used. What we noticed, what I noticed from reading the medical history, is the language barrier, the impossibility of complete understanding (…)* (Croatia).

*The most important topic, which considers us every day in our work, is the topic of language. On one side, many patients speak a language, which is known by someone, maybe from the nursing staff. But it is so, that you cannot treat people so good, as you would if there was no communication barrier. And it is pity, if there is no interpreter available. Of course, we have some co-workers, that are listed as interpreters but it is not that they always can come and help for example with patient information. And it restricts the cooperation between the nursing staff and patients massively* (Germany).

Language barriers were noted as a limitation to efficient and effective treatment of minorities, especially when time and personal constraints exist. Due to a lack of time and communication difficulties, the special needs of minority patients are not properly appreciated, leading to worse health outcomes. The interviewees in Slovenia pointed out that the lack of command of the patient’s language is a barrier not only during individual examination, when an interpreter can be present, but during the entire stay in the hospital, which affects the outcome of the treatment.

According to information provided by our interviewees, seldom is there a regulated or prepared approach to this issue. Healthcare professionals have to rely on improvised unsatisfactory procedures (Table 2). These encompass the use of *ad hoc* interpreters or family members. The use of *ad hoc* interpreters from hospital staff was presented as a regular procedure in most interviews. However, as Slovenian respondents pointed out, with interpreters from the patient’s family, medical professionals often find that perhaps a small portion of the instructions are translated correctly. Several respondents described situations such as that in which an Albanian female patient who did not speak Slovenian relied on her husband as the interpreter. The interviewees were talking about patriarchal relations in the community and their potential impact on adequacy of translation. Especially problematic in this context are translations of intimate health-related facts, e.g., during gynecological examinations.

**Table 2:** Identified challenges in communication and solutions to language barriers in Croatia, Germany, Poland, and Slovenia.

	Challenges identified by interviewees	Currently used solutions
Croatia	Lack of interpreters Lack of translated hospital documents	*ad hoc* interpreters Family members External interpreters Translators employed in hospital Online translation instruments Non-verbal communication
Germany	Lack of interpreters	*ad hoc* interpreters Telephone service Printed documents in several languages Non-verbal communication
Poland	Lack of interpreters Lack of translated hospital documents	*ad hoc* interpreters External interpreters Informal interpreters; available also by telephone Family members, friends; available also by telephone Online translation instruments Non-verbal communication
Slovenia	Lack of interpreters	*ad hoc* interpreters Family members Multilingual manuals for the communication during medical examination Trained medical interpreters Non-verbal communication

According to Polish interviewees, interpreters are sought on an *ad hoc* basis from among the staff or other patients. There were also no informed consent forms available in the patient’s language. Two respondents discussed this issue in detail, in the context of the potential legal responsibility of medical workers when they act as *ad hoc* translators. Respondents from Croatia and Poland mentioned the possibility of providing an interpreter from a consulate or embassy.

Professionals and patients use also grassroot creative solutions, such as phone calls or assistance of informal interpreters. Polish respondents also pointed out to alternative forms of communication if no interpreter was present, for example gestures or Google Translate. Healthcare professionals in Poland mentioned such diverse practices as taking the consent form home and getting it translated by an official translator or just using gestures to show the patient where to sign. They acknowledged the ineffectiveness of the above-mentioned solutions and also the ethical concerns associated with them, i.e., confidentiality of information or quality of informed consent. At the same time, they perceived those measures as satisfactory, emphasizing the role of empathy and “goodwill.” They were rather unsympathetic to the idea of employing interpreters on a regular basis, given the financial situation of Polish hospitals. Use of an online translation engine was also presented as a possibility for communication by respondents from Croatia.

*We had a situation with several migrants, who were brought in by the police were frostbitten and diabetic and so on, so we managed with google translate (…)* (Croatia).

Two German respondents pointed out written documents in several languages that are available to healthcare professionals and can help with communication, e.g., through patient information. In one case, these documents are, according to the respondent, easily available to healthcare staff in the hospital and frequently used. The second respondent mentioned that although these documents are prepared, they are not easily available, and for example, she does not know where to find or how to access them.

Similarly, in Slovenia, there are multilingual manuals to support health workers such as for the Albanian, Arabic, Chinese, Farsi, Russian, French, and Ukrainian languages, but none of the respondents knew about them or intended to use them because they do not have time to browse through them as they need to act quickly and efficiently.

In Germany, only one interlocutor mentioned the use of a telephone service provided by a professional translation agency, which is often contacted to provide translations.

In Slovenia, trained medical interpreters usually work on a voluntary basis and accompany and interpret for people from different language-related backgrounds, which is why they must be sensitive to cultural differences.

*As a volunteer and the only female translator in town, I translate for women at the gynecologist and for children from Arabic into Slovenian, and sometimes I experience impatience and misunderstandings. Some doctors and nurses are unfriendly and roll their eyes and say, “What is it, who came?” Maybe they are stressed or overworked, but they could still be kinder. We agree on the translation about a day before the examination, and just before the examination, I meet the woman in front of the hospital entrance. The nurse knows before the examination that I will be there, so I do the administrative things for the patient with her. During the translation, I stand somewhere in the back, if the patient allows it. The women usually ask for a female doctor, but in emergencies, they also allow a male doctor. I have also assisted in births where I had emergency access to the operating room in case the patients panicked. Sometimes I translate after the delivery, when the woman is already in the ward, and when she is discharged from the hospital. A few times I translated during vaccinations for children and once I accompanied a man for a check-up, but he did not want me to go into the dispensary. I was interested in a full-time job at the hospital, but they did not have the funds* (Slovenia).

### 3.5. Religion and belief

According to interviewees in all four countries, healthcare facilities generally respect the religious needs of patients and hospital staff. Specific issues, such as availability of prayer rooms, respect for religiously motivated dietary needs of patients or treatment of particular religious groups were mentioned in the interviews (Table 3).

**Table 3:** Issues concerning religion and belief in provision of healthcare identified by respondents during the interviews.

	Issues identified by the interviewees
Croatia	Availability of prayer rooms
	Religious issues in diagnostics and treatment
	Respect for dietary needs of the patients
	Treatment of Jehova’s Witnesses
Germany	Influence of religious beliefs on treatment
	Treatment of Jehova’s Witnesses
	Perceived discrimination on grounds of religious beliefs
Poland	Availability of prayer rooms
	Respect for dietary needs of the patients
	Treatment of Jehova’s Witnesses
Slovenia	Availability of prayer rooms
	Respect for dietary needs of the patients
	Treatment of Jehova’s Witnesses
	Ritual circumcision of boys

Since the Catholic religion is predominant in Croatia, Poland, and Slovenia the satisfaction of religious needs of members of this religion, both patients and staff, was mentioned in most interviews. In Croatia, patients in most hospitals have a prayer room where masses are held regularly or occasionally and are seen by a priest when needed. Clergy from other denominations are also available. Visits by an Orthodox priest and a priest for patients of the Islamic faith is also possible if patients express such a wish. The religious needs of employees of the Islamic faith are also respected. Interviewees in Poland pointed to issues related to the functioning of the hospital rather than individual contact with the patients. They referred to the lack of chapels for prayer for non-Catholics and pastoral care provided for Roman Catholics on a regular basis and for other religions on request. Interviewees pointed out that a patient can fulfill the requirements of his or her religion as much as possible in the hospital, e.g., praying in the room, without disorganizing its work. This meant that staff declared that they would try to create conditions for fulfilling religious practices to the extent possible. According to Slovenian interviewees, all hospitals in Slovenia are able and willing to take care of the religious needs of the patients. Most of them have a special room, a Catholic chapel, where religious services are held regularly for patients and medical staff, and some have a meditation park in front of the hospital. If the patient needs a religious ritual of another denomination, they call a priest from the list of other churches. Some hospitals, e.g., oncological ones, even allow New Age rituals if they do not harm the patients – for example, they would not allow the lighting of incense in the hospital or the anointing with certain resins – but they allow decorating the walls in the rooms with religious images.

With regard to religious issues in diagnostics and treatment, interviewees from Croatia provided information that for women of the Islamic faith, the rule is that a female healthcare worker participates in the procedures, and if this is not possible, a compromise solution is found in which a male member of the woman’s family is present. Other medical procedures that may conflict with the patient’s religious affiliation include autopsy.

*(…) one of the situations is, which are rare, but they happen, for example, when asking for an autopsy for the deceased, where in certain religions the autopsy is not acceptable (…). However, there are legal norms of the Republic of Croatia. There is an absolute effort to meet people whenever possible, whenever the diagnosis is clear, whenever we can determine what happened and so on. It exists, but I say these are rare cases, where an autopsy must be performed* (Croatia).

In Germany, interviewees concentrated rather on cultural differences, which, in combination with language barriers, lead to limitation of healthcare and worse treatment. According to their statements, religious topics are less visible in the case of patients with European backgrounds, and more present in the case of patients originating from other backgrounds, i.e., patients originating from predominantly Muslim countries. A lack of trust and confidence on the part of Muslim patients that they would be treated well and not discriminated against was also mentioned; therefore, some patients felt discriminated against for no reason, according to some interviewees.

*There is a cultural barrier with Muslim patients. There is mistrust in communication. It is on both sides or at least on the side of the patients. Mistrust. They come here with mistrust. There are nurses that are less sensitive (to patients’ needs) or do not understand them. And so, arise conflicts from the very beginning and this follows in the whole process* (Germany).

In this context it should also be mentioned that religious beliefs of Jehovah’s Witnesses preventing certain medical treatments, e.g., blood transfusions, influence the level of provided healthcare.

Interview partners in Slovenia mentioned that medical personnel are used to make cultural adaptations in regions with more Roma, such as southern Slovenia. One of such adaptations is taking care of the newborn by the hospital so that the mother can leave the hospital to participate in family rites. They return and pick up the newborn after about a day. According to an interviewed doctor, Roma women usually do not breastfeed, so hospital staff takes care of the infant. In describing this custom, the interviewee did not clearly distinguish between religious and customary issues.

Interviewees also pointed out to the topic of dietary needs of patients. In Croatia, hospitals respect religiously based special dietary restrictions and are able to accommodate them when necessary. A couple of interviewees from Poland stated that diets adapted to individual needs are more and more available in hospitals. The interviewed healthcare professionals understood diet mainly as the absence of certain ingredients, e.g., pork, and not as a diet that complied with kosher or halal rules. Such a diet – excluding only certain ingredients, in their opinion – could be provided to the patient. However, their approach to religious practices and convictions, including diet, varied, depending on their opinion regarding its potential interference with treatment. It depended on the kind of disease. In this context, interviewees expressed a caring attitude:

*At the ward I’m working for, the priority is the diet that we recommend in the hospital. It means she does not have to eat meat, thank God. But, what do they eat during Ramadan? Ok, for sure we will not force anything on her, but we will convince her to regular eating for sure, we will recommend her nutrition in relation to that she has a severe disease, as it is in our ward, and convince her that, I think, that proper diet and nutrition is important in our diseases (…) She can eat at night, but she will be hungry the whole day, then she will fill up, she will have flatulence, abdominal pains, save God, diarrhea, vomits, and it overlaps with the intensive treatment she receives at us, so, it is a big mess. We can keep her on T.P.N as well. Do not Ramadan and Koran take it into consideration? I do not know (…) So, I think that one can try to reason with her, somehow, and say that it is her life and Allah would not like her to die. I do not know. I hypothesize because I have not had such a situation* (Poland).

As for special diets, Slovenian respondents indicated that no hospital offers religiously prepared meals, but several menus are available and patients can choose what they want to eat.

Further issue during the interviews was the question of religiously motivated needs in medical treatment. The respondents in all four countries pointed out to the special situation of Jehovah’s Witnesses. In Croatia, medical staff is familiar with the attitude of Jehovah’s Witness members toward refusing blood transfusions during treatment. One hospital organized workshops attended by surgeons to promote alternative treatment for members of this religious minority. Another hospital has a protocol for such cases that was, among other things, the result of good cooperation with the Jehovah’s Witness Association in that city. The interviewees mentioned the opinion of their colleagues according to which when such patients are in life threatening situations, they are willing to perform a transfusion, knowing full well that litigation might ensue. A particular challenge in relation to this religious minority is how to deal with the treatment of life-threatening illnesses in minors.

*Likewise, we ourselves discussed the way to approach and save the lives of such persons, who, God forbid, would die in a traffic accident and require transfusion treatment, and I must admit that each of my colleagues said that they would rather be sued than refuse to save life to that person* (Croatia).

In Poland, some interviewees were conflicted with regard to the treatment of Jehovah’s witnesses who refuse blood and blood-derived preparations transfusion:

*(…) on one hand, a patient can do everything because it is his life, his body, on the other hand, I’m a doctor, then, am I to look at somebody dying in my arms?* (Poland).

Thus, they pointed out, among other things, to the lack of detailed guidelines for dealing with blood transfusions in the case of those patients, including decisions regarding minors. Also, situations in which a patient consents to a transfusion but the family is not be informed were described, along with creative ways of meeting such a request, like omitting this issue in the information card, but not in the medical documentation.

*Law is law, life is life. It is written “it should be,” not even that it is a duty, just that it should be. So, no one will break off your head if it is not written. That’s the truth. So, nothing will happen* (Poland).

*I heard about such a case, generally, a patient was receiving a blood transfusion, but the blood bag was covered with a black bag and the whole IV drip was covered so that blood dripping was not seen so that the family did not see* (Poland).

In Slovenia, physicians accomodate to the special needs of Jehovah’s Witnesses and treat them without transfusion; although some anesthesiologists have objected on conscience grounds and refused to treat them because it is contrary to medical ethics. Moreover, during the interviews in Slovenia, the topic of ritual circumcision of boys for religious reasons and without medical indication has been raised. One respondent, a surgeon, emphasized that such procedure has been unacceptable in Slovenia since 2012 and is not performed in the public healthcare system for legal and ethical reasons.

### 3.6. Diversity training

Visible from interviews in all four countries is lack of specific training or educational opportunities for healthcare professionals to improve understanding, communication, and treatment of minority patients. It was observed that the challenge of lack of training in cultural sensitivity goes back to medical and health faculties. For example, Polish healthcare professionals observed that almost no time was devoted to such classes during their education, except for courses on patient rights and one university course on intercultural obstetrics reported by an interviewee. Most commonly noted in Croatia, Poland, and Slovenia were trainings on patients’ rights organized in hospitals and workplaces (Table 4).

**Table 4:** Forms of diversity training identified by interviewees.

	Possibilities of diversity training
Croatia	In-hospital training on patients’ rights
	Training in communication skills
	Informal consultations
Germany	Informal consultations
	Irregular courses on diversity competency
Poland	In-hospital training on patients’ rights
	Possibility of commercial diversity courses
Slovenia	In-hospital training on patients’ rights
	Irregular courses on diversity competency

In Croatia, institutional support manifests itself through internal training of newly hired staff on the organization and workflow of the hospital, including patients’ rights and existing protocols. Some hospitals organize training to improve communication skills. There are also opportunities for employees to learn through internal channels within the organization as well as in different organizational units, through consultation with the committee or using specific protocols. In Germany, the majority of respondents were not aware of any specific training or hospital internal documents that provide guidelines for the appropriate treatment of representatives of minority groups. To counteract this shortcoming, respondents mentioned informal measures to address diversity challenges such as an advisory group created as a private initiative by a group of health professionals with an immigrant background or special courses offered for healthcare professionals who contact persons of diverse sexual orientation or gender identity.

*Feelings of strangeness and insecurity are present on both sides. We are feeling insecure in our encounters with migrants but they also feel insecure. They can overcome their insecurity through learning the language, through adjustment to our structures. We can overcome our insecurity by training in intercultural competence. By intercultural opening (…). We need to re-approach and re-define our course of conduct with minority groups* (Germany).

One healthcare professional in Poland pointed out that diversity courses were available on a commercial basis and paid for out of the participant’s own pocket. Moreover, interviewees in Poland spoke both of the necessity of such training courses and their uselessness, as patient rights are a sufficient guarantee of equal treatment.

*There is actually such training, but, in my experience, it is private training, where you have to take the initiative yourself to attend it, there is no training that, for example, a hospital or a unit of some kind would offer, or require you to attend (…)* (Poland).

The interviewees declared that there were no dedicated procedures or regulations on diverse patients in their hospitals. However, two medical doctors referred to the Medical Code of Ethics or the Hippocratic Oath:

*(…) but in the Hippocratic Oath, it is written that you are to treat a patient regardless of his origin, skin color,* etc. (Poland).

*And the Code of Ethics is based partly on the Hippocratic Oath, in which it stands, that I shall not differentiate my help because of nationality or gender or – I think Hippocrates perhaps did not take into consideration transgenderism – but I think it comes from that* (Poland).

In Slovenia, diversity training is not mandatory, and hospitals use their discretion in determining whether or not to require attendance at cultural competency and healthcare training sessions that present examples of best practices. Healthcare workers have limited financial resources and do not have enough time to attend all forms of cultural competency training that are offered from time to time. Some physicians participate in intercultural education voluntarily.

*Some medical professionals try to be as conscious as they can of their own responses to the wide variety of people they encounter and strive to be as impartial as they can when resolving a patient’s issue. The majority, however, have unconscious biases, stereotypes, and other personal beliefs that influence how they treat patients. As far as dealing with people from other cultures, we usually act on the principle of “adapt, get things done, and swim”* (Slovenia).

### 3.7. Proposed solutions

With regard to measures to improve the situation, the interviewees in Croatia, Germany, and Slovenia pointed out the need for training in dealing with patients from minority groups.

*There is space for improvement in the form of perhaps greater awareness among employees that there are more vulnerable groups, who may not self-identify as vulnerable. That we pay more attention to them, that we suspect that they are vulnerable and that we ask them instead of them asking us* (Croatia).

*In Germany (…) we are not at a bad position if you consider the awareness of the problem. But we are not so far, that we can say that we have in our nation characterized by migration, we have achieved everything in the healthcare sector. We are far away from it* (Germany).

Such training should be provided by both state administration and by individual healthcare institutions and offered to all healthcare professionals, including nursing staff. Such education should already begin at the university level not only during the professional career. Training should not only be conducted by specialized training staff or other healthcare professionals but provided with the participation of patients from minority groups. To improve the situation of equal access to healthcare, several other measures need to be implemented. Proposed was the introduction of special guides or attendants, who could improve inter-language and intercultural communication. Also, networking activities between healthcare professionals could lead to mutual help, exchange of experiences, and best-practice examples.

*It would be good on a local level to have a person or a point with pooled competencies, which can inform and facilitate access and which one could contact in case of missing cultural competencies or in case of cultural difficulties* (Germany).

Better availability of professional translation was also mentioned as well as the provision of manuals and guidelines for healthcare professionals and information sheets for patients in their native languages. A higher number of healthcare employees from other countries was proposed as a measure to increase diversity competency. The confirmation of cultural competence of individual physicians and nurses should occur already during the recruitment process and appointment to work in a hospital.

Polish professionals also declare the need for diversity knowledge, competencies, and more generally, raising, the diversity awareness among medical staff. They propose to include relevant training in university curricula. Although they have ambiguous attitudes to training at workplaces, some would welcome them. Their main proposals were very practical and, importantly, low-cost. They include an interpreter available on the telephone, some guidelines concerning transgender patients’ treatment, and providing materials for patients. It seems, that in the current situation of Polish healthcare, they would perceive initiatives such as those proposed by Croatian, German and Slovenian professionals as a waste of scarce resources that could be directed to some more urgent issues. One of the interviewees, who would welcome such training, proposed the following:

*It seems to me, that the thing which could be done, is defining that we have certain groups, concerning whom staff should be trained, working out, on a sort of statutory, national, generally legal level, a pile of money, a healthcare facility should receive money for this kind of training and later, it seems to me, it can be on the very individual level of every hospital or primary healthcare facility how exactly the money from this fund will be distributed* (Poland).

## 4. Discussion

Healthcare is one of the most important sectors that must recognize the specific needs of citizens in order to provide quality services. Lack of understanding and cultural or language barriers create disparities in access to health care between minority representatives and the general population. Such divergences overlay with gaps described in the literature on the topic, which include lack of understanding of patients’ ethnic and cultural models, communication difficulties, limited health literacy, different expectations, and behaviors when seeking treatment, and assumptions and biases about certain groups (32). Accommodating patients’ diverse backgrounds leads to higher numbers of patients treated, better health status, higher levels of trust in the health care system, and greater satisfaction with care (33). However, lack of awareness of the challenges associated with social diversity, the inadequacy of existing healthcare structures, and individual attitudes of health professionals toward minority groups exacerbate healthcare access disparities and worsen healthcare outcomes (34).

The results of the interviews from Croatia, Germany, Poland, and Slovenia show that discriminatory behaviors potentially play a role in the equal treatment of minority patients. Previously, discrimination-related disparities in access to health care were reported in several countries (35–37). Results of the QUALICOPC study 2011–2013 conducted in countries of the European Union show the high variation of perceived discrimination (38). According to these, 7% of the respondents declared instances of discrimination. The highest percentage of perceived discrimination was noted in Sweden (12.8%), and the lowest in Luxemburg (1.4%). Three countries from our investigation, according to the data collected within QUALICOPC study 2011–2013, had low or medium scores of discrimination: Germany (2.8%), Slovenia (6.4%), and Poland (8.6%) (38). Perceived discrimination is a factor limiting access to healthcare (39), through diminished trust and satisfaction with the healthcare system and subsequently lower rates of utilization of healthcare services (34, 37, 40).

Coping with language barriers has been mentioned by interviewees as one of the major factors preventing the provision of equal medical treatment, especially for patients with the first generation migration background. Minority language speakers often face barriers in the utilization of healthcare services (41). These encompass the need to use *ad hoc* interpreters, the reduced number of services offered to minority language speakers, and negative attitudes from the side of healthcare professionals towards patients (42, 43). Lack of common language for communication leads to the limitation of patients’ health and well-being through the diminished quality of service (44). *Ad hoc* solutions regarding interpretation raise ethical concerns with regard to the quality of translation, professional confidentiality, and communication in a triangular relationship between patient, interpreter, and physician (45).

The lack of appropriate policies and organizational support toward equal treatment of minority patients in healthcare institutions was raised by interviewees. In all four countries, research findings have shown the importance of awareness of cultural diversity and cultural competence, which are associated with gaps in health systems. Diversity competency of healthcare organizations can be achieved through several instruments, such as diversity policies, provision of specialized cultural interpreters, and translation of materials for patients. These elements have been indicated by the interviewed healthcare professionals as missing. The organization of a dedicated service for intercultural mediation can contribute to an improvement for patients in navigating administrative procedures or through the provision of information on the patient’s rights and the availability of healthcare services (46, 47). The diversity competence of healthcare practitioners can be improved with specific strategies provided both by the healthcare system and healthcare organizations (48). Among the primary strategies are cultural competence training and professional development interventions. Application of these strategies leads to the improvement of cultural awareness, knowledge, and skills among healthcare professionals and has a positive impact on practitioners’ attitudes, behavior, and confidence (49–51). It can also have positive outcomes for patient satisfaction and trust (52, 53). Organizational readiness to implement clear mission goals and declarations towards diversity competence can also have a positive impact on the provision of better health treatment for representatives of minorities (54); however, it needs to be met with a focused and systematic approach, e.g., through strategic planning, dedicated resources, or specific recruitment practices (55). Such organizational commitment to diversity competency was also postulated by several interviewees.

With regard to the study results, it can be stated that the interview partners from the four countries studied largely agreed in their answers. Nevertheless, there were some differences, of which two in particular stood out. Polish professionals mentioned that underfunding of the healthcare system is the main barrier to accessing healthcare. This is not surprising, as Poland is one of the European countries with the lowest percentage of health care expenditure by the government (56). In addition, there are systemic barriers due to a high rate of out-of-pocket spending by patients and explicit rationing (57). A second difference concerned the group of people who were considered disadvantaged. In this context, Croatian professionals mentioned, among others, persons with alternative live-styles, people in rural areas and chronically ill people. Croatian and German interviewees emphasized that women are to be regarded as disadvantaged, while Slovenian interviewees mentioned Roma, women from Kosovo and Muslim women, among others. The emphasis on different groups is not surprising, because due to the different cultural and political circumstances it can be assumed that different groups of people are disadvantaged. This fact shows that different minorities can be affected by discrimination and exclusion from adequate healthcare. For example, people with a differing sexual orientation or identity in Europe continue to experience significant limitation of their human rights, discrimination and restrictions regarding their healthcare (58). People with a migrant background are also significantly disadvantaged in Europe (59), whereby in Europe a belief in a certain cultural superiority may lead to the formation of racist tendencies (60).

To establish healthcare services that are better equipped to address the needs of patients from social minority groups, systemic interventions that encompass all levels of healthcare systems are necessary. It implies an ethical obligation to address the grounds for health disparities and to ameliorate inequality in access to healthcare for disadvantaged and vulnerable groups. This can occur on several levels: from the macro level of healthcare systems, through the intermediate level of healthcare institutions, to the micro level of individual interactions between patients and healthcare professionals. Thus, interventions need to encompass all these three levels: from policy making and provision of resources to healthcare, through better orientation of healthcare institutions for specific challenges in access to healthcare, to development of individual attitudes and skills of healthcare professionals (61, 62). The results of the interviews show that the respondents specifically stress better adjustment to existing challenges on the level of healthcare institutions, or the national level as it is in Poland, and on the individual level of healthcare practitioners. Therefore, specific mechanisms should be adapted in order to improve the situation. (i) Provision of healthcare is conditioned by healthcare policy. Therefore, structural improvements in healthcare policy need to be implemented. These encompass legislation that specifically addresses and reduces normative barriers in accessing healthcare as well as provision of sufficient financial support for healthcare institutions. (ii) Healthcare institutions need to recognize the importance of internal guidelines for improvement of diversity competency and implement them into their day-to-day functioning. Such guidelines should describe procedures in specific situations and be easily accessible to every healthcare professional. (iii) Inclusion of inter-cultural mediators in order to support understanding of trans-cultural exchange between patients and healthcare professionals. (iv) Use of professional interpreters in order to avoid miscommunication or ethical challenges connected to engagement of *ad hoc* translators. (v) Introduction of administrative support for migrants and refugees and integration of representatives of minority groups into healthcare staff. (vi) Improvement of diversity competency for healthcare professionals through education, starting in medical training and continuing throughout the duration of their careers. Such training can increase sensitivity to ethical issues connected to the question of equal access to healthcare, especially in the case of the most vulnerable groups.

The results of our research have also implications for further investigations of the topic. A better understanding of challenges in accessing healthcare for representatives of minority groups should continue. It should encompass different perspectives, including patients, healthcare professionals, and representatives of healthcare institutions. Empirical research into the translation of institutional policies into practical patient care would be of particular interest.

The question remains whether and to what extent the results of our study can be transferred to other countries. Considering the fact that three of the countries we investigated have medium discrimination scores with regard to the QUALICOPC study (38), it can be assumed that there are still numerous countries in the European Union alone where similar problems of diversity awareness exist. However, discrimination in its many facets is unfortunately a worldwide phenomenon and currently shows an increasing trend (63). It can be assumed that the ways in which minority groups experience discrimination in different countries and health systems may be weighted differently. It is also possible that the relevance of discrimination is assessed differently depending on the cultural background and thus the need for countermeasures is also assessed differently. Since the four countries we studied, which differ culturally but are not entirely culturally disparate, show a relatively uniform picture with regard to diversity awareness, it might be possible, with great caution, to transfer the results to other, countries of the EU, at least in part. Ultimately, however, we see a need for further research here regarding the rest of the world.

## 5. Limitations

Limitations of this research must be considered while analyzing its findings. The results of the interviews cannot be applied to the entire healthcare system or to all health professionals in the countries under investigation due to the sample size of only 39 interviewees. With only 7 to 12 interviews with health professionals per country studied, the results cannot be easily generalized. Furthermore, it is possible that our focus on questions about discrimination and limited access to healthcare could have led to a certain bias on the part of the interviewees. However, these limitations do not fundamentally call the results into question. It is in the nature of qualitative research with the use of interviews that particular aspects might be emphasized by the interview partners. These aspects, however, are otherwise often overlooked and are made visible by the research process. Furthermore, the inclusion of medical experts from various specialties and professional levels, such as doctors, nurses, health care managers, and medical interpreters, makes it possible to examine various viewpoints. The sample size permits a thorough examination of various viewpoints as well as each respondent’s particular concerns. As a result, the findings require triangulation in a separate research.

Moreover, since in many cases healthcare institutions were gatekeepers, some participants certainly gave socially desirable answers, that is, they tried to present themselves, their colleagues, and the institution in a better light. It could also be the case of participants who were contacted independently. Additionally, persons who decided to participate in this research could be those who are more interested in diversity topics and therefore their diversity awareness was higher than in the general population of medical professionals, which could also impact the data. Also, the circumstances of conducting the interviews, i.e., a large number of researchers, combining face-to-face and online techniques could have affected data collection. Differing number of interviewees from countries participating in the research also point out to response bias. All attempts have been made to include similar number of interview partners from all four countries in the time intended for research.

The method of thematic analysis itself also has limitations: the coding system developed is subjective and thus subject to interpretation. Furthermore, only the occurrence of certain themes is identified and not the relations in which the themes stand. Nevertheless, thematic analysis has the advantage of identifying very diverse and broad themes and can thus be a valuable starting point for further research on particular topics.

## 6. Conclusion

In this research, several disadvantaged groups in health systems were identified in all four countries. These consist particularly of persons, who are poor, cannot navigate the healthcare system without the help of others, or are socially stigmatized, e.g., persons of sexual minority groups. The results show that systemic discrimination and barriers may relate to inadequate legal and administrative regulations that interfere with addressing patients’ special healthcare needs, as well as internal hospital culture due in part to the lack of specific cultural diversity awareness training and medical ethics.

Although the majority of healthcare professionals did not perceive any direct systemic exclusion of minority groups, they recalled several instances of indirect discrimination or situations in which they did not have culturally specific knowledge about how to communicate with disadvantaged groups. Also, they recalled acts of discrimination on the individual level, which stemmed from individual attitudes toward minorities, which ranged from the rare extreme presence of silent racism and denigration of certain groups to a basic lack of cultural competence and empathy, but each country is specific in this regard. Language barriers, lack of diversity skills, and lack of support from health care providers in the form of medical interpreters or training were cited as major challenges. Insufficient resources mean that awareness of patients’ individual needs cannot be fully achieved, which could lead to unwanted health outcomes.

In order to improve these shortcomings: (i) healthcare professionals should be supported by improved legislation and funding to remove normative and financial barriers in access to healthcare; (ii) internal guidelines should include aspects of diversity awareness and diversity competency and their compliance should be ensured; (iii) intercultural mediators should be employed to improve trans-cultural exchange; (iv) professional translators should be employed in all steps of medical care; (v) administrative support for migrants and refugees should be introduced or optimized; and (vi) diversity competency should be improved through training. For this, healthcare professionals who themselves belong to a minority group could also come forward and thus be effective as trans-cultural ambassadors in the health system.

More research is needed on the topic of this study: the different types of discrimination against minorities in different countries and cultural spaces should be researched. Furthermore, it would be important to identify solutions for different types of discrimination worldwide. These could serve as a blueprint for other countries where such discrimination still takes place. Moreover, it would be important to better understand the relationship between systemic and individual types of discrimination. This could reveal at which levels it is most effective to implement anti-discriminatory measures.

## Data availability statement

The original contributions presented in the study are included in the article/Supplementary material, further inquiries can be directed to the corresponding author.

## Ethics statement

The studies involving human participants were reviewed and approved by The Ethics Committee for biomedical research at the Faculty of Medicine, University of Rijeka, Research Ethics Committee at the Ulm University, The Institutional Review Board of The Human Research Ethics Committee at the University of Ljubljana, University of Warsaw Rector’s Committee for the Ethics of Research Involving Human Participants. The patients/participants provided their written informed consent to participate in this study.

## Author contributions

FS, AM, PŁ, and ZZ-S: conceptualization and funding acquisition. MR, MO, RD, KB, ITG, TS-E, MN, and AC: investigation. MR and MO: writing—original draft. MR, MO, RD, KB, AC, ITG, TS-E, MN, FS, AM, PŁ, and ZZ-S: review and editing. All authors contributed to the article and approved the submitted version.

## Funding

The research was conducted within the scope of the project “Healthcare as a Public Space: Social Integration and Social Diversity in the Context of Access to Healthcare in Europe.” The project “Healthcare as a Public Space: Social Integration and Social Diversity in the Context of Access to Healthcare in Europe” is financially supported by the Humanities in the European Research Area (HERA) Joint Research Programme under HERA Public Spaces Culture and Integration in Europe Programme (Hera.2.029)^1^ which is co-funded by German Federal Ministry of Education and Research (BMBF); National Science Centre, Poland (project no. 2018/28/Z/HS1/00554); Croatian Academy of Sciences and Arts; Slovenian Ministry of Education, Science and Sport, and European Commission through Horizon 2020 (grant agreement no 769478).

## Conflict of interest

The authors declare that the research was conducted in the absence of any commercial or financial relationships that could be construed as a potential conflict of interest.

## Publisher’s note

All claims expressed in this article are solely those of the authors and do not necessarily represent those of their affiliated organizations, or those of the publisher, the editors and the reviewers. Any product that may be evaluated in this article, or claim that may be made by its manufacturer, is not guaranteed or endorsed by the publisher.

## Footnotes

^1^ www.heranet.info
